# Hepatic resection for hepatocellular carcinoma in the octogenarian: is it justified?

**DOI:** 10.18632/aging.101854

**Published:** 2019-03-13

**Authors:** Chao-Wei Lee, Kun-Ming Chan, Hsin-I Tsai, Yi-Chung Hsieh, Cheng-Yu Lin, Yung-Chia Kuo, Heng-Yuan Hsu, Ming-Chin Yu

**Affiliations:** 1 Division of General Surgery, Department of Surgery, Chang Gung Memorial Hospital, Linkou Medical Center, Guishan, Taoyuan, Taiwan; 2 College of Medicine, Chang Gung University, Guishan, Taoyuan, Taiwan; 3 Graduate Institute of Clinical Medical Sciences, Chang Gung University, Guishan, Taoyuan, Taiwan; 4 Department of Anesthesiology, Chang Gung Memorial Hospital, Linkou Medical Center, Guishan, Taoyuan, Taiwan; 5 Division of Hepatology, Department of Hepatogastroenterology, Chang Gung Memorial Hospital, Linkou Medical Center, Guishan, Taoyuan, Taiwan; 6 Division of Gastroenterology, Department of Hepatogastroenterology, Chang Gung Memorial Hospital, Linkou Medical Center, Guishan, Taoyuan, Taiwan; 7 Department of Hematology-Oncology, Chang Gung Memorial Hospital, Linkou Medical Center, Guishan, Taoyuan, Taiwan

**Keywords:** hepatic resection, hepatocellular carcinoma, octogenarian, elderly

## Abstract

Liver resection is a standard treatment for hepatocellular carcinoma (HCC). The purpose of this study was to investigate the clinicopathological characteristics and long-term outcomes of octogenarians with HCC treated with liver resection. Records of patients who underwent liver resection for HCC were reviewed, and patients older and younger than 80 years were compared. There were 77 patients 80 years of age or older and 3,309 younger than 80 years. Hepatitis C virus infection was the most common etiology among the octogenarians (43.1%), followed by non-viral causes (37.5%). The older group had more co-morbidity but less hepatitis B virus infection and cirrhosis. More than 70% of the non-viral older group had diabetes mellitus, as compared to only 21.6% in the viral older group. The older group had rates of perioperative morbidity, mortality, disease-free survival, and overall survival comparable to the younger group (all *p*>0.1). Multivariate analysis revealed that α-fetoprotein ≥400 ng/mL, tumor size ≥10 cm, and vascular invasion were independent prognostic factors for overall survival in the older patients. These findings demonstrate that liver resection is a justified treatment for HCC in octogenarians, and it can achieve surgical outcomes comparable to those in younger populations.

## Introduction

Hepatocellular carcinoma (HCC) is the second most common cause of cancer-related death in the world [1]. In Taiwan, it causes more than 8000 deaths annually [2]. With increases in the average life expectancy, cancer treatment for super-elderly (more than 80 years of age) patients has become a global issue; however, there are currently no published guidelines for treating octogenarians with HCC. Because of the existing difficulties in making treatment decisions for the super-elderly, surgeons and oncologists may delay standard surgical resections or hesitate to initiate less invasive locoregional therapies.

Kinoshita et al. reported that there are no significant differences in overall survival (OS) or disease-free survival (DFS) between super-elderly and younger HCC patients not indicated for surgical resection [3]. Earlier studies also showed that hepatic resection is reasonable for HCC patients older than 55 or even 70 years of age [4,5]. However, little data is available on the outcomes of octogenarians treated for HCC with liver resection. The aim of the present study was to investigate the clinicopathological features of HCC in the octogenarians and to compare the surgical and oncologic outcomes between octogenarian and younger HCC patients. We also analyzed risk factors for in-hospital mortality and predictors of long-term survival in the octogenarians.

## RESULTS

### Patient demographics

A total of 3,386 HCC patients treated with curative hepatectomy were included in the study. Among them, 77 patients were ≥ 80 years old and were classified as the octogenarian group (O-HCC, 2.3%). The remaining patients were younger than 80 years old and were designated as the younger patient group (Y-HCC, n=3,309, 97.7%). The mean age at diagnosis in the two groups was 83 and 57 years, respectively. The median follow-up time was 39.3 months in the O-HCC group and 45.8 months for the Y-HCC group. Their respective clinical characteristics are summarized in Table 1. As shown, the O-HCC group had significantly more co-morbidities, less hepatitis B virus (HBV) infection, and lower hemoglobin and albumin levels than the Y-HCC group (all *P* < 0.05). While HBV infection accounted for more than 66% of HCC in the Y-HCC group, hepatitis C virus (HCV) infection was the most common etiology in the O-HCC group (n=28; 43.1%). Interestingly, 27 patients (37.5%) in the O-HCC group had neither HBV nor HCV infection, which was significantly higher than in the Y-HCC group (10.6%, *P* <0.001). Furthermore, not listed in the table was our finding that 73.1% of non-viral O-HCC patients had diabetes mellitus (DM), as compared to only 21.6% of viral O-HCC had DM (*P* < 0.001).

**Table 1:** Comparison of clinical characteristics between octogenarians and younger patients with hepatocellular carcinoma.

**Variables^a^**	**Total** **(n=3386, 100%)**	**Age ≥ 80 y** **(n=77, 2.3%)**	**Age** < **80y** **(n=3309, 97.7%)**	**Odds ratio**	***p* value**
**Gender** (Male(%)/Female(%))	2647(78.2)/739(21.8)	58(75.3)/19(24.7)	2589(78.2)/720(21.8)	0.849	0.54
**Comorbidity** (Yes (%))	1034 (31.2)	49 (63.6)	985 (30.5)	3.996	<0.001
**Diabetes Mellitus** (Yes (%))	630 (19)	32 (41.6)	598 (18.5)	3.135	<0.001
**Hypertension** (Yes (%))	486 (30.1)	32 (45.1)	454 (29.5)	1.965	0.005
**ESRD^b^**(Yes (%))	68 (2.1)	4 (5.2)	64 (2.0)	2.714	0.049
**Stroke** (Yes (%))	42 (2.6)	4 (5.6)	38 (2.5)	2.361	0.101
**HBs Ag**^c^(Positive (%))	2018 (65.3)	16 (23.2)	2002 (66.3)	0.154	<0.001
**Hepatitis C virus** (Positive (%))	963 (35.7)	28 (43.1)	935 (35.6)	1.372	0.211
**Non-B Non-C** (Yes (%))	363 (11.2)	27 (37.5)	336 (10.6)	5.061	<0.001
**Child-Pugh Classification** (A(%)/B(%)/C(%))	3156(95.5)/146(4.4)/2(0.1)	72(96.0)/3(4.0)/0(0)	3084(95.5)/143(4.4)/2(0.1)	N.A.^k^	0.961
**Symptoms**^d^(Yes (%))	1370 (40.5)	24 (31.2)	1346 (40.7)	0.660	0.092
**Tumor number**(Single (%))	1354 (87.1)	47 (95.9)	1307 (86.8)	3.578	0.061
**Procedure** (Major^e^ resection(%))	1333 (41.1)	24 (32.4)	1309 (41.3)	0.682	0.124
**ICG-15**^f^(%)^l^	11.09 ± 0.20	11.83 ± 1.34	11.08 ± 0.21	N.A.^k^	0.583
**Hemoglobin** (g/dL)^l^	13.25 ± 0.35	12.05 ± 0.2	13.27 ± 0.04	N.A.^k^	<0.001
**Albumin** (g/dL)^l^	4.03 ± 0.01	3.88 ± 0.06	4.03 ± 0.54	N.A.^k^	0.017
**Platelet** (1000/uL)^l^	178.46 ± 1.44	175.59 ± 7.43	178.53 ± 1.46	N.A.^k^	0.759
**ALT**^g^(U/L)^l^	58.17 ± 1.20	52.81 ± 6.34	58.30 ± 1.22	N.A.^k^	0.491
**Bilirubin total** (mg/dL)^l^	0.93 ± 0.02	0.81 ± 0.12	0.93 ± 0.02	N.A.^k^	0.257
**Alkaline phosphatase** (U/L)^l^	100.9 ± 3.34	92.42 ± 5.31	100.89 ± 3.41	N.A.^k^	0.709
**Pre-op á-fetoprotein**^h^(ng/mL)^l^	8249.4588 ± 1699.2	4497.46 ± 2846.89	8337.31 ± 1737.64	N.A.^k^	0.735
**Pre-op CEA**^i^ (ng/mL)^l^	6.05 ± 1.45	2.83 ± 0.27	6.14 ± 1.49	N.A.^k^	0.713
**Pre-op CA-199**^j^(U/mL)^l^	457.99 ± 265.76	35.92 ± 6.39	470.11 ± 264.1	N.A.^k^	0.781
					

a: only patients with available data were analyzed; b: end-stage renal disease; c: Hepatitis B virus surface antigen; d: HCC with preoperative symptoms include anemia, jaundice, palpable mass, or ascites; e: Major procedures include tri-segmentectomy, right/left lobectomy, and extended right/left lobectomy; f: Indocyanine green retention test at 15 min; g: Alanine aminotransferase; h: preoperative serum α-fetoprotein level; i: preoperative serum carcinoembryonic antigen level; j: preoperative serum carbohydrate antigen19-9 level; k: not applicable or not done; l: mean ±standard error of mean.

There were no significant differences between the two groups with respect to tumor size, encapsulation, capsular invasion, tumor rupture, vascular invasion, daughter nodules, resection margin, or Edmondson-Steiner grade (all *P*>0.05) (Table 2). In the O-HCC group, however, there was significantly less liver cirrhosis than in the Y-HCC group (38.2% *vs.* 53.9%, *P*=0.007).

**Table 2:** Comparison of pathological characteristics between octogenarians and younger patients with hepatocellular carcinoma.

**Variables^a^**	**Total** **(n=3386, 100%)**	**Age ≥ 80 y** **(n=77, 2.3%)**	**Age** < **80y** **(n=3309, 97.7%)**	**Odds ratio**	***p* value**
**Tumor size** (cm)^b^	5.49 ± 0.07	5.91 ± 0.39	4.17 ± 0.7	N.A.^c^	0.368
**Encapsulation** (Yes (%))	2398 (75.7)	60 (80)	2338 (75.6)	1.294	0.376
**Capsular invasion** (Yes (%))	1720 (55.4)	44 (58.7)	1676 (55.4)	1.143	0.57
**Tumor rupture** (Yes (%))	288 (8.7)	11 (14.5)	277 (8.5)	1.81	0.07
**Vascular invasion** (Yes (%))	1166 (36.3)	31 (41.3)	1135 (36.2)	1.243	0.358
**Daughter nodules** (Yes (%))	814 (24.8)	20 (26.3)	794 (24.8)	1.084	0.758
**Resection margin** (Negative (%))	2241 (93.2)	62 (92.5)	2179 (93.2)	0.905	0.832
**Edmondson-Steiner grade** (I and II (%))	1707 (55.6)	39 (52.7)	1668 (55.6)	0.888	0.616
**Cirrhosis** (Yes (%))	1174 (53.5)	29 (38.2)	1745 (53.9)	0.527	0.007
**T stage** **T1** (%) **T2** (%) **T3a/T3b** (%) **T4** (%)	1622 (50.6) 688 (21.5) 515/86 (16.1/2.7) 296(9.2)	36 (49.3) 15 (20.5) 9/3 (12.3/4.1) 11 (13.7)	1586 (50.6) 673 (21.5) 506/83 (16.1/2.6) 286 (9.1)	N.A.^c^	0.578
**N stage** (N1 (%))	36 (1.1)	0 (0)	36 (1.2)	N.A.^c^	0.354
**M stage** (M1 (%))	54 (1.7)	0 (0)	54 (1.7)	N.A.^c^	0.255

a: only patients with available data were analyzed; b: mean ±standard error of mean; c: not applicable.

### Comparison of surgical and oncological outcomes

The outcomes after hepatectomy for HCC are summarized in Table 3. The total complication rate was 42.4% for the entire cohort. There were no significant differences in the complication rate, 30-day mortality rate, in-hospital mortality rate, or mean hospital stay between the O-HCC and Y-HCC groups (all *P* >0.05). To further investigate the differences between younger and older patients, a subgroup of 278 HCC patients operated on by a single surgeon between 2011 and 2015 was analyzed. Among this group, 11 patients (4%) were ≥ 80 years old. After statistical analysis, the most common surgical complication within both groups was ascites requiring diuretics. Post-operative bleeding, surgical site infection, jaundice, and bile leakage rarely occurred in the O-HCC group. However, one patient in the O-HCC group underwent endotracheal intubation and mechanical ventilation due to respiratory failure.

**Table 3:** Complications after hepatectomy for hepatocellular carcinoma.

**Variables**	**Total** **(n=3386, 100%)**		**Age ≥ 80 y** **(n=77, 2.3%)**		**Age** < **80y** **(n=3309, 97.7%)**		***p* value**	
**Any complications** (n (%))	1393(42.4)		46 (63.9)		1336 (41.6)		0.147	
**30-day mortality** (n (%))	61 (1.8)		3 (3.9)		58 (1.8)		0.162	
**In-hospital mortality** (n (%))	97 (2.9)		5 (6.5)		92 (2.8)		0.059	
**Mean hospital stay**(days)^a^	11.25 ± 0.31		14.12 ± 1.89		11.15 ± 0.31		0.102	
**Surgical complications** ^b^		**Total** **(n=278, 100%)**		**Age ≥ 80 y** **(n=11, 4.0%)**		**Age** < **80y** **(n=267, 96.0%)**		***p* value**		
**Wound infection**(n (%))	9 (3.2)		0 (0)		9 (3.4)		0.536	
**Bile leakage** (n (%))	12 (4.3)		0 (0)		12 (4.5)		0.472	
**Post-OP bleeding requiring re-operation** (n (%))	1 (0.4)		0 (0)		1 (0.4)		0.839	
**Ascites requiring diuretics**(n (%))	82 (29.5)		5 (45.5)		77 (28.8)		0.236	
**Jaundice** (n (%))	2 (0.7)		0 (0)		2 (0.7)		0.773	
**Pleural effusion** (n (%))	5 (1.8)		1 (9.1)		4 (1.5)		0.184	
**Respiratory failure requiring intubation** (n (%))	2 (0.7)		1 (9.1)		1 (0.4)		0.078	

a: mean ±standard error of mean; b: analysis performed on a subgroup of patients operated by a single surgeon between 2011 to 2015 (total patients: 278, Age ≥ 80 y: 11).

**Figure 1:**
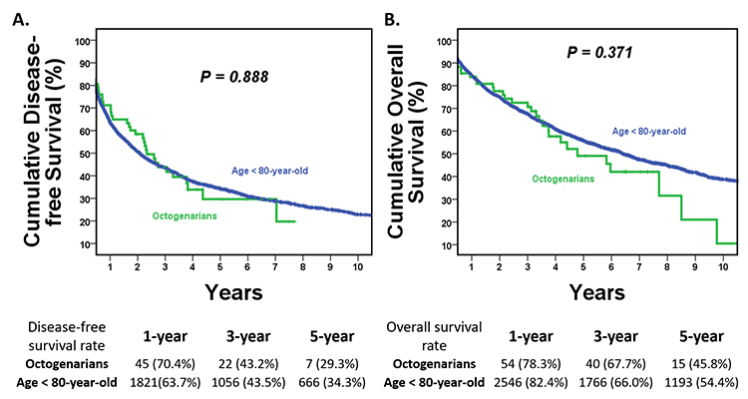
**Kaplan–Meier disease-free survival (DFS) and overall survival (OS) curves for hepatocellular carcinoma treated by hepatectomy in the O-HCC and Y-HCC groups.** (**A**) Disease-free survival curves. The median DFS was 27.9 months in the O-HCC group and 24.9 months in the Y-HCC group (*P*=0.888). The 5-year DFS rate was 29.3% in the O-HCC group and 34.3% in the Y-HCC group (*P* =0.671). (**B**) Overall survival curves. The median OS was comparable between the two groups. The median OS was 57.4 months in the O-HCC group and 77.1 months in the Y-HCC group (*P*=0.371). The 5 year-OS rate was 45.8% in the O-HCC group and 54.4% in the Y-HCC group (*P* =0.379).

The median disease-free survival (DFS) for the entire cohort was 25.1 months (95% CI, 22.7-27.4). There was no significant difference in median DFS between the O-HCC and Y-HCC groups (27.9 *vs.* 24.9 months, *P*=0.888) (Figure 1). The overall survival (OS) for the entire cohort was 76.5 months (95% CI, 69.8-83.2), and the median OS for the two groups were comparable (57.4 *vs.* 77.1 months, *P*=0.371) (Figure 1). The 5-year DFS rate was 29.3% in the O-HCC group and 34.3% in the Y-HCC group (*P* =0.671), while the 5-year OS rate was 45.8% in the O-HCC group and 54.4% in the Y-HCC group (*P* =0.379). To reduce bias related to the prolonged study duration, a subgroup analysis of patients operated on after year 2001 was conducted. The results were comparable to those for the entire cohort, and there was no significant difference in DFS or OS between the two groups (*P*=0.735 and 0.123, respectively). To strengthen the result further, a propensity score matched analysis derived from the entire cohort was performed. Clinical variables including gender, co-morbidity, hepatitis viral status, preoperative hemoglobin, albumin, and cirrhosis were matched between the two groups. Again, the DFS and OS for the two groups were comparable (*P*=0.287 and 0.433, respectively).

### Risks factors for in-hospital mortality in the octogenarians

Five patients (6.5%) in the O-HCC group died in hospital after hepatectomy for HCC. The risk factors for in-hospital mortality were analyzed, and the results are summarized in Table 4. INR >1.4, serum α-fetoprotein >200 ng/mL, major procedure, and major surgical complications were all potential risk factors for in-hospital mortality after hepatectomy for HCC in the octogenarian group (all *P* <0.05). All other clinical variables tested were unrelated to in-hospital mortality.

**Table 4:** Risks factors for in-hospital mortality after hepatectomy for hepatocellular carcinoma in the octogenarian.

**Variables**	**Cases with in-hospital mortality** (%)	**Odds ratio**	***p* value**
**Gender** (male vs. female)	5 (8.6) vs. 0 (0)	N.A.^g^	0.186
**Diabetes mellitus** (yes vs. no)	3 (9.4) vs. 2 (4.4)	2.224	0.387
**Hypertension** (yes vs. no)	2 (6.3) vs. 3 (7.7)	0.813	0.800
**End-stage renal disease** (yes vs. no)	0 (0) vs. 5 (6.8)	N.A.^g^	0.588
**Old stroke** (yes vs. no)	0 (0) vs. 5 (7.5)	N.A.^g^	0.571
**Smoking** (yes vs. no)	1 (7.7) vs. 4 (6.3)	1.250	0.847
**Alcohol** (yes vs. no)	0 (0) vs. 5 (7.1)	N.A.^g^	0.498
**HBs Ag**^a^ (positive vs. negative)	2 (12.5) vs. 2 (3.8)	3.645	0.191
**Hepatitis C virus** (positive vs. negative)	2 (7.1) vs. 2 (5.4)	1.345	0.773
**ICG-15**^b^ (> 10 vs. ≤ 10(%))	2 (7.7) vs. 3 (8.6)	0.889	0.901
**Hemoglobin** (≤ 10 vs. > 10 (g/dL))	1 (7.7) vs. 4 (6.3)	1.250	0.847
**Albumin** (≤ 3.5 vs. > 3.5 (g/dL))	1 (5.0) vs. 3 (5.6)	0.894	0.925
**Platelet** (≤ 100 vs. > 100 (1000/uL))	1 (10.0) vs. 4 (6.0)	1.751	0.630
**INR**^c^(> 1.4 vs. ≤ 1.4)	1 (100) vs. 4 (5.6)	N.A.^g^	<0.001
**ALT**^d^(> 40 vs. ≤ 40 (U/L))	2 (6.5) vs. 3 (6.8)	0.943	0.950
**Bilirubin total** (> 1.5 vs. ≤ 1.5 (mg/dL))	0 (0) vs. 4 (5.6)	N.A.^g^	0.675
**á-fetoprotein** (> 200 vs. ≤ 200 (ng/mL))	5 (21.7) vs. 0 (0)	N.A.^g^	0.001
**Procedure** (major vs. minor (%))^e^	4 (16.7) vs. 1 (2.0)	9.804	0.019
**OP duration** (> 270 vs. ≤ 270 (min))	3 (12.0) vs. 2 (3.9)	3.341	0.182
**Blood loss** (> 800 vs. ≤ 800 (mL))	1 (6.7) vs. 4 (6.7)	1.000	1.000
**Inflow control** (yes vs. no)	3 (6.3) vs. 1 (8.3)	0.733	0.796
**Tumor size** (> 10 vs. ≤ 10 (cm))	0 (0) vs. 5 (7.8)	N.A.^g^	0.316
**Surgical complications** (major vs. minor/none)^f^	5 (25.0) vs. 0 (0)	N.A.^g^	<0.001

a: Hepatitis B virus surface antigen; b: Indocyanine green retention test at 15 min; c: International normalized ratio; d: Alanine aminotransferase; e: Major procedures include tri-segmentectomy, right/left lobectomy, and extended right/left lobectomy; f: Major surgical complications include grade III-IV surgical complications; g: not applicable or not done.

### Prognostic factors for the octogenarians

Cox regression multivariate analysis revealed that only HCV infection and tumor rupture are independently associated with a significantly poorer DFS in the O-HCC group. As shown in Table 5, HCV infection and tumor rupture were respectively associated with a 4.26-fold and 7.36-fold increase in risk of HCC recurrence after hepatectomy in the octogenarians (all *P* < 0.001). Although its impact was not yet statistically significant, O-HCC patients with α-fetoprotein ≥400 ng/mL were also prone to recurrence. On the other hand, serum α-fetoprotein ≥400 ng/mL, tumor size ≥10 cm, tumor rupture, and vascular invasion were all found to be associated with a poorer OS (Table 6, all *P* <0.05). Cox regression multivariate analysis revealed serum α-fetoprotein ≥400 ng/mL, tumor size ≥10 cm, and vascular invasion to be independent poor prognostic factors for poorer OS in the octogenarians (all *P <*0.05).

**Table 5:** Cox regression multivariate analysis for disease-free survival in octogenarian with hepatocellular carcinoma.

**Variables**	**Univariate**					**Multivariate**	
	**Median survival** (months)^a^	***p* value**			**Hazard ratio (95% CI)**		***p* value**
**Gender** (male vs. female)	27.9±5.1 vs. 27.2±7.9	0.934					
**Comorbidity** (yes vs. no)	31.1±3.1 vs. 20.9±17.2	0.599					
**Hepatitis B virus** (yes vs. no)	23.0±10.4 vs. 27.9±3.9	0.746					
**Hepatitis C virus** (yes vs. no)	9.0±4.1 vs. 84.4±33.9	<0.001			4.261 (2.07 ~ 8.79)		<0.001
**á-fetoprotein ≥ 400ng/mL** (yes vs. no)	13.1±7.7 vs. 32.9±6.9	0.055					
**Surgical complications** (yes vs. no)	31.7±11.7 vs. 27.2±5.9	0.459					
**Tumor size ≥ 10cm**(yes vs. no)	7.1±2.6 vs. 31.7±5.5	0.153					
**Encapsulation** (yes vs. no)	27.7±2.9 vs. 32.9±11.2	0.304					
**Tumor rupture** (yes vs. no)	5.5±1.3 vs. 36.6±7.9	<0.001			7.358 (2.98 ~ 18.2)		<0.001
**Vascular invasion** (yes vs. no)	20.9±6.5 vs. 39.4 ±7.9	0.163					
**Daughter nodules** (yes vs. no)	27.9±7.6 vs. 45.3± 12.4	0.402					
**Resection margin** (positive vs. negative)	36.6± 26.2 vs.31.1 ± 3.3	0.848					
**Edmondson-Steiner grade**(I /II vs. III/IV)	31.7±7.2 vs. 26.7±9.0	0.612					
**Cirrhosis** (yes vs. no)	26.3±6.2 vs. 32.9±7.9	0.098					

a: median±standard error of median.

**Table 6:** Cox regression multivariate analysis for overall survival in octogenarian with hepatocellular carcinoma.

**Variables**	**Univariate**					**Multivariate**	
	**Median survival** (months)^a^	***p* value**			**Hazard ratio (95% CI)**		***p* value**
**Gender** (male vs. female)	52.9 ± 17.5 vs. 57.4 ± 10.7	0.871					
**Comorbidity** (yes vs. no)	52.9 ± 7.8 vs. 37.6 ± 28.6	0.686					
**Hepatitis B virus** (yes vs. no)	57.4 ± 27.9 vs. 50.2 ± 21.4	0.472					
**Hepatitis C virus** (yes vs. no)	42.4 ± 16.7 vs. 92.4 ± 15.3	0.259					
**á-fetoprotein ≥ 400ng/mL** (yes vs. no)	14.1 ± 23.5 vs. 71.5 ± 14.4	0.011			2.235 (1.09 ~ 4.57)		0.028
**Surgical complications** (yes vs. no)	57.8 ± 6.8 vs. 59.9 ± 7.8	0.489					
**Tumor size ≥ 10cm**(yes vs. no)	11.3 ± 13.3 vs. 57.4 ± 13.5	0.040			2.304 (1.04 ~ 5.12)		0.041
**Encapsulation** (yes vs. no)	50.2 ± 7.5 vs. 92.4 ± 57.5	0.677					
**Tumor rupture** (yes vs. no)	7.4 ± 9.6 vs. 69.9 ± 12.5	0.001			2.088 (0.79~ 5.51)		0.137
**Vascular invasion** (yes vs. no)	37.6 ± 14.5 vs. 102 ± 22	0.002			2.301 (1.09 ~ 4.88)		0.030
**Daughter nodules** (yes vs. no)	57.3 ± 12.8 vs. 45 ± 15.4	0.286					
**Resection margin** (positive vs. negative)	3.3 ± 1.3 vs. 57.4± 12.7	0.135					
**Edmondson-Steiner grade** (I /II vs. III/IV)	69.9 ± 18.1 vs. 57.4± 15.4	0.514					
**Cirrhosis** (yes vs. no)	57.4 ± 9.6 vs. 50.2 ± 17	0.831					

a: median ±standard error of median.

## DISCUSSION

HCC is endemic in Taiwan with an age-adjusted incidence of 23.3/100,000 per annum, making it the second leading cause of cancer-related death among men and women there [2]. Owing to the aging population, the incidence of HCC in elderly patients has been rising in recent decades; the mean age of HCC patients at first diagnosis has increased from 60 years in the mid-nineties to 70 years in the twenty-first century [6]. The mean expected life expectancy for populations older than 80 years in Taiwan is 9.48 years, long enough to consider anti-cancer treatment [7]. Because physiological processes in elderly patients may differ somewhat from those in younger ones, it is of paramount importance to assess the treatment strategy for this unique group of patients. Importantly, the liver appears to be less influenced by aging than other organs, such as the lungs, heart, and kidneys [8,9]. Livers from octogenarian donors can still be transplanted safely [10]. As a result, we believe that age itself should not be the only factor when considering treatments for octogenarian HCC patients.

In the present study, several distinct clinical-pathological features were observed among the octogenarian HCC patients. First, the incidence of HBV-related HCC was significantly lower among octogenarians than younger HCC populations. Less than 25% of patients in the O-HCC group had HBV infection, as compared to more than 65% of patients in the Y-HCC group. HCV infection, on the other hand, was the most common etiology in the O-HCC group, which is consistent with earlier reports [6,11,12]. One possible explanation is that HBV infections are usually acquired early in a patient’s life due to perinatal transmission, whereas most HCV infections are acquired later in life, and HCV-related carcinogenesis requires a longer time to develop [4,5,12–14]. Notably, nearly 40% of O-HCC patients had neither HBV nor HCV infection, and a large majority of those patients (92.1%) denied habitual alcohol consumption. Carcinogenesis is therefore considered to be related to nonalcoholic steatohepatitis (NASH) [6,15,16]. Furthermore, the significant correlation between DM and non-viral O-HCC suggests that aging, DM and NASH all contribute to the development of HCC in the elderly, though that hypothesis remains to be tested. The O-HCC group also had significantly less liver cirrhosis than the Y-HCC group, possibly due to the fact that NASH contributes to a significant proportion of HCC in the elderly. This implies that cirrhosis is not a prerequisite for the development of HCC in older populations. Aging itself may be sufficient to hepatocarcinogenesis.

Patients in the O-HCC group were also found to have lower preoperative albumin and hemoglobin levels, which reflects their advanced age. Despite the presumption of poorer body reserve, the proportion of patients receiving major liver resections was similar between the O-HCC and Y-HCC groups, indicating that the type of liver surgery should not be altered simply due to advanced age. In contrast to a reported preponderance of females among the older population with HCC [17,18], the majority of our O-HCC patients were males, which may be attributable to the different ethnicity and treatment strategies among different countries or geographic regions.

In the past, the risks of surgical complications and uncertainty about the long-term results have made conservative treatment the preferred approach for elderly HCC patients [19,20]. However, given the improvements in surgical technique, operative instruments, and perioperative care, the treatment strategy for octogenarian patients should be adjusted. Consistent with the report that the Eastern Cooperative Oncology Group (ECOG) score is one of the most important determinants of 3-month postoperative mortality in geriatric cancer patients [21], good performance status before a planned hepatectomy is necessary for elderly HCC patients.

Despite encouraging results, the in-hospital mortality rate among the O-HCC group was slightly higher than among the Y-HCC group. The significant risk factors contributing to in-hospital mortality include INR >1.4, α-fetoprotein >200, major liver resection, and major surgical complications. Because INR, α-fetoprotein, and type of liver resection are inherent patient or tumor factors, the only adjustable variable is the major surgical complications. According to recent studies, major surgical complications are one of the most significant independent factors contributing to early mortality after liver resection for HCC, and it also plays a significant role in promoting tumor recurrence [22,23]. Because super-elderly HCC patients have comparably less physiologic functional reserve, recovery from a serious complication may be more challenging for them than their younger counterparts. As a result, liver surgeons should be meticulous in their effort to avoid major surgical complications after hepatectomy in elderly HCC patients. Barring surgical complications and mortality, these super-elderly patients can enjoy a DFS and OS comparable to those achieved in younger populations!

The present study demonstrated that HCV infection and tumor rupture are the most significant predictors of tumor recurrence after hepatectomy in octogenarians. Because HCV infection is the most common etiology of HCC in octogenarians, and it is usually associated with gradual development of cirrhosis, appropriate antiviral therapy and stringent screening are mandatory for octogenarians suffering from HCV infection [6,19]. Furthermore, application of more effective adjuvant therapy and close postoperative follow up are also indispensable when octogenarian HCC patients are found to have these prognostic factors. Regarding OS, as with general HCC populations, a large tumor size (≥10 cm), high α-fetoprotein (≥400 ng/mL), and vascular invasion remain independent factors predictive of poor OS in octogenarians [24].

Table 7 summarizes the results of several studies examining the efficacy of liver resection in the elderly [4,5,12–14,25–29]. Most of these studies agree that liver resection can be performed in well-selected elderly patients. However, few studies defined elderly patients as older than 80 years old. Ours is by far the largest single cohort of octogenarian HCC patients whose outcomes after liver resections have been examined.

**Table 7:** Literature review on the surgical outcomes of elderly patients with hepatocellular carcinoma undergoing hepatectomy.

**Year**	**Author**	**Cutoff** **age** **(years)**	**Elderly patients (%)**	**Complication rate (Major) (%)** **(*P*-value** ^a^**)**	**Hospital mortality (**%) **(*P*-value** ^a^**)**	**DFS** **(*P-*value** ^a^**)**	**OS** **(*P*-value** ^a^**)**	**Special remarks**
1987	Ezaki *et al.* [28]	>65	37(24)	NS	NS	NA	NS	Preoperative liver function, histology, and control of bleeding determined results.
1988	Yanaga *et al.* [29]	≥65	27(17.5)	NA	40.7 (<0.05)	NA	NA	Sepsis (72.7%) is the most common cause of hospital mortality. Limited resection in cirrhosis.
1990	Fortner *et al.* [27]^b^	>64	90(19.9)	NA	11.1^c^	NA	NA	Extended right lobectomy should be performed in selected cases.
1999	Wu *et al.* [26]	≥80	21(8.0)	14.3 (0.88)	0 (0.51)	NS (0.15)	NS (0.46)	Liver resection for HCC is justified in selected octogenarians, with short- and long-term results comparable to those of younger patients.
2008	Kondo *et al.* [14]	≥70	109(34.2)	NS	NS	NS	NS	The only difference in postoperative complications was the frequency of pneumonia observed in elders.
2009	Huang *et al.* [12]	≥70	67(20.0)	9.0(0.220)	1.5 (>0.999)	NS (0.157)	↑ (0.017)	Hepatectomy for selected elderly patients with HCC have a better curative effect compared with younger patients.
2011	Portolani *et al.* [4]	≥70	175(38.8)	16.0 (0.85)	3.4 (0.79)	NS(>0.05)	NS(>0.05)	Liver resection is a valid option in the elderly. However, major resections must be reserved for selected cases.
2012	Su *et al.* [5]	>55	700(65.2)	NA	NA	NS(0.75)	NS(0.71)	A Propensity Score Matching Analysis; Age is not a risk factor to determine the prognosis of patients with HCC who underwent resection.
2015	Xu *et al.* [25]	>55	205(45.5)	NA	NA	↓ (<0.05)	↓ (0.04)	Age is a risk factor to determine the prognosis of patients with HCC.
2015	Ha *et al.* [13]	>40	247(87.6)	NA	NA	NS(0.218)	↑(0.032)	Young patients have more aggressive clinicopathologic features and poor prognosis.
2019	Lee *et al.*	≥80	77 (2.3)	63.9^d^ (0.147)	6.5 (0.059)	NS (0.888)	NS (0.371)	Liver resection is a justified treatment modality for HCC in the octogenarians. DM and NASH may contribute to the development of HCC in the elderly populations.

NS - statistically not significant, NA - not applicable or not done, ↓ - significantly worse compared with that of younger age group. a: Compared with the younger age group; b: Included hepatectomy for non-HCC liver diseases; c: Mortality rate for major hepatic resection; d: Total complication rate.

Despite promising results, the present study has several limitations. First, incomplete or missing clinical data are inevitable when retrospectively reviewing medical records. Second, the treatment strategy for HCC may have evolved over the study period, which could potentially influence the study results. Studies with shorter recruiting duration may address this issue; however, the sample size for octogenarians would not be sufficient to obtain statistical significance. Third, surgeon bias may be a confounding factor leading to less homogenous results. Further studies are still required to provide additional power to our findings.

## CONCLUSIONS

Our findings indicate that HCV infection is the most common etiology of HCC in octogenarians, and that in the absence of viral hepatitis, DM together with NASH may contribute to the development of HCC in the elderly. In well-selected octogenarians with acceptable liver function, an adequate future liver remnant, and a good ECOG score, liver resection is a justified treatment modality for HCC, and surgical outcomes comparable to those in younger populations can be achieved. Further studies are warranted to validate our findings.

## MATERIALS AND METHODS

### Study population

We retrospectively reviewed HCC patients treated with curative hepatectomy by our surgical team at Chang Gung Memorial Hospital (CGMH) between 1986 and 2015. The patients were divided into a younger patient group (Y-HCC group; age < 80 years old) and an octogenarian group (O-HCC group; ≥ 80 years old) according to their age at first diagnosis. Patients with distant metastases, patients who underwent non-curative intent hepatectomy, or those whose detailed medical records were unavailable were excluded from the study. A total of 3,386 patients were ultimately enrolled. The demographics, perioperative and survival data were reviewed. Our primary study endpoints were surgical complications and in-hospital mortality. Secondary endpoints were disease relapse and long-term survival. The study end date was December 31, 2015. Tumor staging was based on the American Joint Committee on Cancer (AJCC) TNM staging system for HCC [30].

### Diagnosis and preoperative evaluation

Diagnoses of HCC were established based on definitive pathological examination; typical image features on dynamic computed tomography (CT), magnetic resonance imaging (MRI), arterial angiography; and/or a serum α-fetoprotein level >200 ng/ml. Resection criteria included appropriate liver function, adequate future liver remnant, no distant metastasis to other organs, and an absence of tumor thrombi in the main portal vein. Liver function was routinely evaluated using the Child-Pugh classification and indocyanine green retention test at 15 minutes (ICG-15). In our institute, an ICG-15 ≤ 10% was necessary for major liver resection (three or more liver segments) [22,23]. In addition, an ECOG score of 0 to 1 was a prerequisite at the time the operation was to be performed [31]. For octogenarians, echocardiography and pulmonary function tests were routinely performed before the operation. High risk patients were routinely assessed by cardiologists and anesthesiologists to determine the operative risk. For diabetic patients, blood sugar was maintained below 200 mg/dl during the perioperative period. Blood pressure was controlled at an appropriate level and anticoagulant/antiplatelet medications were adjusted or held as indicated before the operation.

### Surgery, postoperative management, and follow-up

All operations were conducted by the same surgical team in the Department of General Surgery. The liver texture, tumor location and extent, and vascular patency were routinely evaluated by manual palpation and intraoperative ultrasonography. Hilar lymph node dissection was performed if enlarged lymph nodes were detected [32]. Liver parenchyma transection was performed using either the clamp crush technique, cavitron ultrasonic surgical aspirator (CUSA) and/or other energy devices. Inflow control was applied based on the individual patient's condition and the surgeon's preference. Bleeding and bile leakage were checked meticulously at the end of the surgery.

Patients were sent to either intensive care units or ordinary wards for postoperative care. Enteral nutrition was resumed as early as possible. Blood products and albumin were transfused whenever necessary to maintain adequate serum albumin levels (usually > 3.0 g/dL), urine output, and ascites control. Prophylactic antibiotics were discontinued 24 hours after surgery if there were no signs of infection. Hemograms and biochemical data were checked routinely on postoperative days 2 and 7. All patients were followed-up with triphasic CT and measurement of serum α-fetoprotein levels every 3 to 6 months after hospital discharge.

### Definitions

Major liver resection was defined as resection of three or more liver segments including tri-segmentectomy, right/left lobectomy, and extended right/left lobectomy [33]. Complications after hepatectomy included wound infections, bile leakage, bleeding requiring re-intervention, ascites requiring diuretics, jaundice, pleural effusion, respiratory failure requiring intubation, and mortality. Patients who tested positive for bacteria in the wound discharge were considered to have a wound infection. Bile leakage was defined as one or more of the following: 1) drainage of bile from the abdominal wound or drain; 2) intraabdominal collection of bile confirmed at the time of reoperation or percutaneous drainage; and 3) cholangiographic evidence of biliary leakage or stricture [34]. Ascites was defined as daily ascites fluid drainage exceeding 500 ml and/or more than grade 2 ascites on ultrasonography or clinical assessment showing a moderately symmetrical distension of the abdomen [35,36]. Diuretics, starting with an aldosterone antagonist (spironolactone) and then a loop diuretic (furosemide), were administered in a stepwise manner in these circumstances. Clinically symptomatic fluid collection in the pleural space that was confirmed by either X-ray or ultrasound was considered to be postoperative pleural effusion. As for jaundice, a persistently abnormal serum bilirubin level on postoperative day 7 and/or no significant improvement compared to day 2 was classified as postoperative jaundice.

Recurrence was defined as the appearance of characteristic image findings during regular postoperative radiologic examinations. Early recurrence was defined as recurrence within two years of the initial curative operation. DFS was calculated from the date of surgery to the date of the first documented clinical disease recurrence. OS was defined as the time elapsing from the date of diagnosis to either the date of death or the date of last contact. Cases with surgical mortality, defined as death within one month of surgery, were excluded from the survival analyses.

### Statistical analysis

Statistical analysis was performed using IBM SPSS Statistics 21 (IBM Corporation, Software Group, Somers, NY, USA). Student’s t test or Mann-Whitney U test was used to analyze continuous variables. Fisher’s exact test or Pearson’s χ^2^ test was used to analyze categorical data. Kaplan-Meier analysis was used to determine the OS and DFS. The log-rank test and Cox proportional hazard regression multivariate analysis were adopted to determine prognostic significance of clinicopathological variables in the octogenarian group. Values of *P* < 0.05 in two-sided tests were considered significant.

### Ethics approval and consent to participate

This study was approved by the Institutional Review Boards (CGMH IRB No: 201600359B0) of Chang Gung Memorial Hospital (CGMH).

### Availability of data and materials

All data generated or analyzed during the study are included in this published article. Raw data may be requested from the authors with the permission of the institution.
